# Parental cancer: communication, daily life changes and psychosocial support: a qualitative study of adolescents and young adults who experienced parental cancer during adolescence

**DOI:** 10.1186/s40359-025-03396-3

**Published:** 2025-09-17

**Authors:** Wiebke Geertz, Laura Inhestern, Corinna Bergelt

**Affiliations:** 1 Department of Medical Psychology, University Medical Centre Hamburg-Eppendorf (UKE), Martinistraße 52, 20246 Hamburg, Germany; 2 Department of Medical Psychology, University Medicine Greifswald, Greifswald, Germany

**Keywords:** Adolescents, Parental cancer, Psychosocial support, Psycho-oncology

## Abstract

**Background:**

A parent’s cancer diagnosis affects all family members. Adolescents in particular seem to be vulnerable to distress and changes associated with the cancer diagnosis. This study aims to gather information about the experiences of adolescents whose parents have cancer. We analysed communication and changes in daily life due to the parents’ illness, as well as preferences for professional psychosocial support.

**Methods:**

Semistructured interviews were conducted with 17 participants aged 15–35 years, who had experienced a parent’s cancer in their youth. The analysis was conducted through content analysis, and the results are reported according to the COREQ criteria.

**Results:**

The data analysis yielded the following key themes: cancer-specific communication, the impact of cancer on the family and on adolescents, preferences for professional psychosocial support and advice for adolescents with a parent with cancer. According to the interviewees, most families talked about their parents’ cancer. However, the extent and content varied greatly. The most frequently mentioned topics were facts about cancer and treatment. Information was frequently shared in the event of a medical appointment or intervention being scheduled. The emotional impact of the illness was rarely discussed. Both positive and negative effects on role shifts and dynamics within the family were reported, e.g., greater family cohesion or more conflicts. With regard to support needs, the majority of participants considered psychosocial support services to be useful, whether in individual sessions, with family or in groups. The optimal approach would be a combination of conversations and activities, such as joint excursions or creative activities. Furthermore, participants expressed the view that parents should make use of support services and that teachers and schools should play a more active role. They would advise other affected individuals to engage in open dialogue with parents and to seek support if necessary.

**Conclusion:**

The study shows that a parent’s cancer diagnosis in adolescence can significantly impact various aspects of life, and that young people want to be involved in communication about their parents’ illness. The findings suggest personalised psychosocial support, especially through peer groups, is effective in supporting adolescents with a parent who has cancer.

**Supplementary Information:**

The online version contains supplementary material available at 10.1186/s40359-025-03396-3.

## Background

Adolescence is widely regarded as a critical period for the development of human potential. During this phase, an individual acquires the foundation for health and wellbeing later in life in terms of physical, cognitive, emotional, social and economic resources [1]. Adolescence is defined as the period between late childhood and early adulthood (typically characterised as the time span between the ages of 10 and 19 years). During this developmental stage, adolescents undergo significant biological changes and social role transitions [2]. In addition to physical and cognitive development, social and emotional development are also important for this phase. Worries about being normal, the increasing influence of peer groups, and the stronger desire for independence and learning to regulate emotions are just a few examples of the developmental challenges faced by adolescents [2].

When a parent has cancer, children and adolescents face additional challenges as they grow up. The age and developmental stage of the child or adolescent influences their capacity to cope with familial issues. The majority of children have the capacity to adapt to new circumstances and, in an ideal scenario, to mature accordingly. Nevertheless, many children react to stressful situations by attempting to give the impression that all is well.

It can be difficult for children and young people to talk honestly about their feelings when other family members are present, as they are often considerate of their parents’ feelings [3]. This has the potential to contribute to an underestimation of the emotional strain on children [4].

Adolescents appear to be particularly vulnerable to the distress and changes associated with a parental cancer diagnosis [5, 6]. Potential stressors include being separated from the sick parent for longer periods of time because of hospital stays, taking on extra domestic chores and responsibilities for younger brothers and sisters, and coping with the fear of losing a parent [7].

Previous research has explored helpful aspects for adolescents facing the dual burdens of parental illness and age-specific challenges. Social support, listening, practical support, communication about the disease and its treatment, and participation in normal life experiences were identified as helpful factors for adolescents with a parent with cancer [8]. Adolescents also consider coping strategies such as ‘time away’, ‘distraction’, ‘informing school’ and ‘trying to maintain normalcy’ to be beneficial [9].

In addition to these personal strategies, there are various options for professional psychosocial support for children and adolescents with a parent who has cancer, e.g., web-based support interventions [10–12], psychosocial counselling for families [13] or manualized interventions such as CLIMB [14]. While previous research has focused mainly on the general needs of adolescent offspring of cancer patients and their support needs in relation to family and friends, little is known about their preferences for professional psychosocial support.

## Methods

The reporting of this article follows the Consolidated Criteria for Reporting Qualitative Research [15]. The study was approved by the local ethics committee (Local Psychological Ethics Committee at the Centre for Psychosocial Medicine, University Medical Centre Hamburg-Eppendorf; LPEK-0257).

### Objectives of this study

This study concentrated on the views of adolescents and young adults who had received a parental cancer diagnosis during their own adolescence and their opinions on the most appropriate types of professional support. The following research questions were investigated:

1. What are the perspectives of adolescents and young adults who have been affected by a parent’s cancer diagnosis during their own adolescence on communication within and outside the family and on the changes in everyday life and in the family that are associated with their parents’ illness?
2. What type of professional psychosocial support would they consider helpful and suitable for adolescents in this situation?
3. What advice would they pass on to adolescents who have recently become affected by a parent’s cancer diagnosis?

### Participants

In-depth interviews were conducted with a group of adolescents and young adults (aged 15–35 years) who had experienced parental cancer during their own adolescence (aged 10–19 years).

#### Inclusion and exclusion criteria

All cancer types affecting the ill parent and disease course were included. We excluded cases where the parent had been diagnosed within the past two years and participants with cognitive limitations or insufficient German language skills. We selected this time interval with the objective of ensuring that the interviewees had already gained experience with their parents’ cancer, which they could reflect upon.

#### Sampling and sample size

Participants were recruited consecutively via handouts handed out among student assistants at the University Medical Centre Hamburg and their friends and in cancer counselling centres throughout northern Germany. The handouts were circulated further via email to institutions working in the field of psychosocial support for families with parental cancer, as listed by the German Association of Psychosocial Oncology. The initial target sample size was set at 15 former adolescents; however, through purposeful sampling techniques aimed at diversifying demographics, we successfully interviewed 17 individuals. Following a consecutive sampling strategy and referring to existing literature demonstrating that saturation can be reached with a limited number (9–17) of interviews [16], we stopped inclusion when no further aspects were mentioned. One candidate did not fully meet the required inclusion criteria and so could not be included in the project.

### Data collection

#### Procedure

Interested adolescents and young adults were invited to contact the study team, who provided them with detailed information about the study. Following the acquisition of written consent from the study participants or, in the case of minors, their parents, WG and a second researcher conducted semistructured qualitative telephone interviews from September 2021 to June 2022. Both researchers are qualified psychologists with extensive experience in conducting qualitative studies. The study participants had the opportunity to take part in the interview either in person or by telephone. All the interviewees chose to participate via telephone. A semistructured interview guideline, which was developed previously, was used. The interview guidelines were developed on the basis of the research team’s professional experience and relevant literature [17]. The interview guide was tested on a volunteer interviewee to ensure the structure and comprehensibility of the questions and to make any necessary amendments. As no adaptations were needed, the pretest interview was included in the data analyses. Each interview started with background information on the research project and information on data protection. An overview of the interview guidelines is provided in Table 1. The interviews were recorded, and field notes were taken during the interviews. The audio files were transcribed verbatim via the f4transkript software V.7 (www.audiotranskription.de). Transcripts were not returned to participants for comments or corrections.

**Table 1:** Overview of the interview topics relevant to the analysis

Topic	Central questions ^a^
Sociodemographic data	e.g. age, education, parent’s cancer diagnosis
Experiences regarding communication	Who told you about the disease?
	How did you talk about it within the family?
	Where did you get your information from (parents, books, internet…)?
	Did you receive all necessary information?
	Was there an opportunity to talk to doctors or accompany the ill parent to doctors’ appointments or visit in hospital?
Changes due to the parents’ illness	What changes occurred within the family and in daily life due to the illness?
	How did the family cope with it?
	What was helpful?
Psychosocial Support	Did you receive any kind of professional support?
	Which family member made use of support offers?
	What type of support would you have wanted?
	What would be an ideal professional psychosocial support offer for adolescents?
Current situation	How did the parents’ illness influence your life thus far?
	What advice would you give to children and adolescents when a parent hat cancer?
Ending of the interview with the possibility for participants to ask questions and to add further thoughts or experiences.	

^a^ Prompting questions and, if necessary, detailed questions were asked

### Data analysis

The interview transcripts were analysed according to the principles of Mayring’s qualitative content analysis [18]. WG generated categories both deductively (on the basis of the interview guidelines and theoretical considerations) and inductively (from transcripts) via MAXQDA Version 22, a qualitative data analysis software. LI was consulted during the generation of the coding system and during the coding process in the event of unclear assignments of codes. The themes were reviewed and refined through a critical dialogue with all authors. The quotations were translated from German to English by WG and LI. The analysis of sociodemographic variables was conducted utilising descriptive statistics with the software IBM SPSS Statistics V.27.

## Results

The results of our research are presented in accordance with the research questions, which relate to communication, changes due to the parent’s cancer and thoughts on professional psychosocial support offers. Tables 2 and 3 present an overview of the themes and subthemes related to communication and changes.

**Table 2:** Communication and changes - superordinate themes, themes, and subthemes and example quotes

Themes	Subthemes	Example Quotes (Participant Code)
**Communication**		
Structural aspects		
Conversational partner	Parents	“At that time, it simply helped to talk about it. Just with relatives, so with grandma and grandpa or with my mother, unfortunately my sister was never truly the person who likes to talk about such topics. Or sometimes I just talked to my closest friends, I have two, with whom I still talk about it when something is bothering me.” (I15, male, parent with palliative cancer)
	Siblings	
	Other family members	
	Friends	
	Teacher	
	Medical staff	
	Others affected	
Frequency	Often/regularly	“Whenever there was news, we talked about it. For example, when it was certain that it would be operated or if there was any news or something. I also asked from time to time how it looked, so nothing was kept secret or avoided.” (I12, male, parent survived cancer) “We never talked about it like that and I knew that if I brought it up, there would not be much of a response. That is why I have always avoided it a bit.” (I07, female, parent with relapse/chronic cancer)
	Before or after doctors’ appointments or treatment	
	Seldom, rarely, hardly ever	
	Own insecurity	
	Fear of burdening parents	
	Parents withheld information	
	Parents did not want to talk	
Content aspects		
Topics	Medical information	“Well, there were moments when we also talked about the fact that it was difficult and how we were coping, especially because I was … I was getting worse and worse with this whole situation and then we also started to talk about feelings, but I think at first it was more about the facts.” (I04, female, parent survived cancer)
	Diagnosis	
	Prognosis	
	Treatment	
	Death	
	Changes in family/individual life	
	Feelings and worries	
Sources of information about the cancer	Parents	“One of my aunts is a doctor, not an oncologist, but I think if you have studied medicine, you understand a bit about it. In addition, she also helped me, so she always told me that if I had any questions about what was happening or any medical questions in general, then I should just ask her.” (I12, male, parent survived cancer)
	Other family members (often with a medical background)	
	Books, brochures	
	Internet	
	Medical staff	
**Impact of cancer**		
**Family**		
Financially	Less money because of lower income of the sick parent	“Everything froze a bit and you could not truly plan things. In addition, then there was somehow, I think, a relatively long period of waiting: “What happens now?” (I06, female, parent died of cancer)
Uncertainty/not be able to make plans		
Presence of other caregiver	Grandparents	“There was a lot of support during the phase when my father was in the palliative care ward. I know that an aunt of mine took time off completely during that time and was at home with us.” (I11, female, parent died of cancer)
	Other family members	
Living situation	Ill parent moved out	“And because Mum had just had chemo six months before, she was in hospital with Corona for two or three months, during which time I was alone at home.” (I09, female, parent with cancer in remission)
	Ill parent moved home after working and living in another city due to cancer	
	Absence of ill parent because of hospital stays	
Family dynamics	Role shifts Between parents Parentification/young carer	“I’m not the oldest, but I was the one who took on most of the responsibility, and in fact, from one moment to the next, you’re no longer a child, that is how it felt. You take on the role of a mother somehow for your siblings, you take on other roles in relation to your father, I had the feeling, that I was the link that my mother had been before.” (I16, female, parent died of cancer)
	Changes regarding the parents Physically Psychologically	“In fact, I remember very well that my mother was also quite burdened at that time. She had lost a lot of weight, for example, and looked a bit worn out, and my father anyway.” (I04, female, parent survived cancer)
	Within family interaction Consideration for parents Joint activities More visits/more contact Stronger family cohesion Conflicts More conflicts Less conflicts	“And above all, he then was at home every day and we had to be very considerate of him, which limited the things we could do in the living room, for example, because my father always wanted to have his peace and quiet and always expressed that, so I spent a lot of time in my room or outside doing sports or with friends.” (I15, male, parent with palliative cancer)
**Adolescents** Communication	More communication with others Lying to friends/keeping the cancer secret	“Yes, that was a bit of a difficult thing, which I also experienced as difficult at the time, because my father did not want us to talk about it with others. In addition, that was sometimes weird for me, for example, I know that a friend, a good friend, asked me about it and I got into the conflict because he explicitly said beforehand that he did not want us to talk about it. (…) And I felt under pressure and yes, that was the case, that it was not communicated openly.” (I06, female, parent died of cancer)
Emotional changes	Fears and worries Other family members could get cancer too I myself could get cancer Recurrence To infect the parents with SARS-CoV-2 virus Of death of the parents	“So I know that it scared me a lot. I hadn’t truly had any contact with the subject of cancer before. That is for sure. In addition, I was in such a phase of transition myself, with a new phase in my life, so to speak, and I still remember that it was just a lot, so that on top of it.” (I04, female, parent survived cancer)
	Suppression	“So that has certainly changed. Before that, we were both appreciative and grateful, so we always had that, but I think even more so now. It has definitely changed that we appreciate each other more and such beautiful experiences and holidays, especially after such a hard time.” (I09, female, parent with cancer in remission)
	Distress	
	Eating Disorder	
	Sadness	
	Gratefulness	
	Taking on more responsibilities	
	Getting stronger	
School	Difficulties in school	
	School as resource and distraction	
Lifestyle	Healthy diet	“So now I’m kind of paying attention to my diet because I’m afraid that there could somehow be factors that can somehow influence the development of cancer. So I think I’d better watch out and leave out things that are not that good, so that I can somehow protect myself from it.” (I03, female, parent died of cancer)
	Pay attention to hygiene	
	Regular cancer screening	
Leisure Time	Meeting friends for distraction	“When my mother was ill, there were certainly dates or parties I would have liked to go to, especially for sports, but when I know “oh, someone was ill, someone is ill”, I’d rather not go, before I would have said, well, I will just go, it is not so bad.” (I10 female, parent survived cancer)
	Hobbies as a resource	
	Spending more time at home	
	Minimise contact to reduce risk of COVID-19 infection	
	No meeting friends at home (risk of infecting parents)	
Educational path	Choosing city/location near home Moving back home Deliberately moving further away to create distance	“Well, I was newly studying at university, I interrupted it because I was just too far away from home and wanted to be there. At least when she was diagnosed that she would not live much longer.” (I16, female, parent died of cancer)

**Table 3:** Wish for psychosocial support and advice for others - superordinate themes, themes, subthemes and example quotes

Themes	Subthemes	Example Quotes (Participant Code)
**Professional psychosocial support**		
No need for psychosocial support		“I Just didn’t need it.” (I17, male, parent survived cancer)
Structural Aspects		
Access path	Flyer	“Somehow proactively get in touch like “Oh, there is cancer here as a diagnosis”, and then maybe, hmmm how do you reach children best, of course you think of social networks now, but maybe also just an initiative letter or something like that.” (I02, male, parent with palliative cancer)
	Posters	
	Parents as gatekeepers	
	Internet	
	Medical staff	
Location	Hospital/Counselling centre	“However, simply that there is someone who could perhaps be approached, i.e., a neutral person. That there is simply an offer in the hospital where it is then said: “Come here if you want and spend the time”. Where different families and children can come.” (I16, female, parent died of cancer)
	Neutral location	
	School	
Instructor	Psychologist or medical Staff	“That somehow, one or two psychologists meet with a group of young people, either by telephone or depending on the situation. That you can exchange information with others who have had the same experience. Because, for example, I do not know anyone in my circle who has experienced similar things.” (I09, female, parent with cancer in remission)
	Teacher	
	An external/neutral person (not a family member or relative)	
Setting	Individual	“I have heard about systemic therapy, that would have been something I would have participated in and I think it would have done us good to talk about it together in the family and maybe also work on other issues that might come up. In addition, personally, I have always had a lot of trust in teachers, so there are a few situations in my life where I have had intensive conversations with teachers and they have helped me and I think teachers could have contributed to that, because as a student you are at school for 6-7-8 hours a day.” (I14, male, parent survived cancer)
	Family	
	Peer-group	
	Consultation hours	
	Open offer	
	Mix (individual, family and group setting)	
	Platform to establish contact to other affected people	
	Internet platform with videos	
	Brochures and books	
Topic/content		“So maybe you could also have regular talks, of course it would be good if someone accompanied you a little bit, someone professional. In addition, that one gets information about the disease, about cancer and understands a little bit where it comes from and what the consequences are for the ill person.” (I13, female, parent survived cancer) “Playing group games together, playing music together, painting Easter eggs together outside, I do not know, having an activity that you enjoy, moving around a bit, exercise always helps me to get thoughts out of my head. That you do something like that together.” (I10, female, parent survived cancer)
Talks	Information about cancer	
	Emotion	
	Coping strategies	
	Bereavement support	
Activities	Excursions	
	Creative elements	
	Games	
	Sports	
	Music	
Additional wishes	Family rounds in hospital	“So I think it would have been good if there had been some kind of immediate support, with brochures or something, to get an idea of how the course of the disease can be, I think that would have been helpful. In addition, at the time when it was clear that my father was going to have an operation I think it would have been helpful if there had been some kind of talks or something, maybe I would not have got thus far off track as I did in the end.” (I04, female, parent survived cancer)
	Low threshold	
	Support from the beginning	
	More school/teacher involvement	
	Honest and clear information about prognosis	
	Parents would seek support themselves	
**Advice for adolescents with a parent with cancer**	Take time to process it	“That they should definitely seek help if they need it. Do not hide your worries in any case. Talk to someone, whether it is friends, family or someone else, and keep being busy, move a lot, so that you do not just think about it.” (I16, female, parent died of cancer) “So I think what’s important in any case is to know that you’re not alone, but that it affects a lot of people and above all that it is okay that you suffer from it or that it affects you, because you might think sometimes that since you are not the person with the illness at that moment, you do not have to make a fuss about it, but in the end it also makes a lot of difference to the people who are around the ill person.” (I13, female, parent survived cancer)
	Try to see positive things	
	Do not hide your own worries and needs	
	Motivate parents to seek support	
	Seek support yourself	
	Talk about it with family/friends/teachers	
	Ask questions	
	Spend time with parent/keep in touch	

### Participant characteristics

A total of 17 individuals, comprising 11 females (65%) and 6 males (35%), participated in the study. The average interview duration was 35 min (range: 15–70 min). At the time of the interview, the age of the participants ranged from 15 to 35 years. The mean age of patients at their parents’ initial diagnosis was 15 years (SD 3.3 years). Two participants had experienced the loss of a parent at a younger age than 16, and three had lost their parent before they turned 20. In two cases, siblings participated in the interviews. This resulted in a total of 17 former adolescents from 15 different families being interviewed. The most prevalent diagnoses were breast cancer (29%), followed by leukaemia (18%) and skin, pancreatic, and colon cancer (each 12%). Table 4 provides an overview of the sample’s sociodemographic characteristics.

**Table 4:** Sociodemographic data of *N* = 17 participants

	Total (*N* = 17)	
	mean	SD/range
Age in years	23.9	6.58/15–35
Age at initial diagnosis of the parent	14.8	3.29/6–19
Time since initial diagnosis	9.12	4.72/3–16

	*n*	%^d^
**Sex**		
Female	11	65
Male	6	35
**Diseased parent during adolescence** ^a^		
Mother	8	47
Father	9	35
**Cancer diagnosis**		
Breast cancer	5	29
Leukaemia	3	18
Pancreatic cancer	2	12
Skin cancer	2	12
Colon cancer	2	12
Other	3	18
**Current state of disease**		
Deceased	7	41
Long term survival ^b^	6	35
Palliative	2	12
Relapse/chronic	2	12
**Professional psychosocial support**		
None	9	53
Psychosocial counselling (individual setting)	4	24
Psychotherapy ^c^	4	24
**Level of education**		
Still attending school	4	24
12 or more years of school education	7	41
University degree	6	35
**Number of siblings**		
None	2	12
One	12	71
Two	3	18

^a^ Three participants had both parents diagnosed with cancer, but the second parents’ diagnosis occurred when they were already young adults. The interview study focused solely on parental disease during adolescence

^b^ Survival longer than 5 years

^c^ Two of the participants partook in psychotherapy later in the process

^d^ Percentages have been rounded up

### Communication

With regard to communication about parental illness, the study participants mentioned different structural aspects, such as how often they communicated and who they communicated with, as well as content-related aspects, such as medical information, changes in family life and worries. In retrospect, the majority of participants felt that they had been adequately informed about their parents’ diagnosis during adolescence. However, some expressed the sentiment that crucial information had been withheld from them, and they suspected that their parents had wanted to spare them. In the vast majority of families, parents take the initiative to inform their children about the cancer diagnosis themselves.

*I understood that she was ill and that it could be dangerous*,* but I never knew the smaller things. (…) I just did not know some things and I do not know if I truly wanted to know them*,* but in retrospect*,* I was missing a lot of information. (I03*,* female*,* parent died of cancer)*

In many cases, conversations about cancer within the family occurred around the time of a doctor’s appointment or treatment. In families where communication was limited, the interviewees indicated that their parents either withheld information or were reluctant to discuss their illness. In addition, sometimes the young people themselves did not dare to start a conversation because they felt insecure or did not want to burden their parents.

Parents, other family members and friends were frequently identified as individuals with whom the interviewees discussed their parents’ cancer diagnosis, whereas teachers, psychologists and other healthcare professionals were mentioned less frequently. The most common topics discussed among family members in relation to cancer were diagnosis, prognosis and treatment. Topics such as personal emotions, family dynamics or the prospect of bereavement have rarely been addressed in this context.

*I would say it was mostly when something was coming up*,* so that we were always prepared (…) and so that we could understand why the person was not doing so well and that we could look after each other. However*,* otherwise*,* I would say there was not so much talk about whether it was a burden on us or not. (I13*,* female*,* parent survived cancer).*

### Cancer impact on the family

The reported changes that cancer brought to the family were very diverse and related to various factors, such as the family’s financial situation, the well-being of the parents, and above all, the family’s dynamics. One of the changes in the family that the interviewees experienced due to the parental cancer disease was the presence of other caregivers, e.g., grandparents, taking care tasks from the parents. Further changes were reported, including financial insecurities and difficulties in planning ahead, as well as extended periods of absence from the ill parent due to hospitalisation. The majority of the observed changes were associated with alterations in family dynamics. The interviewees described significant role shifts at the parental level, as well as behavioural changes in themselves. These included considerations such as avoiding burdening or infecting the sick parent with a virus and taking on more household chores than usual.

*I brought food and kind of*,* yes*,* got blankets when she wanted a blanket or straightened the pillows (…). I kind of helped her when she wanted to go to the toilet. That kind of thing. In addition*,* just*,* yes*,* helped around the house*,* hanging out the washing*,* doing the dishes and stuff like that. (I05*,* female*,* parent died of cancer)*

The participants reported a range of physical and psychological changes, including mood swings and weight loss, in both parents, particularly in the affected parent. The alterations in familial interactions were described in a heterogeneous manner. A number of participants reported that there had been a decrease in the number of arguments within the family unit and that the family had been committed to preserving harmonious relationships. In other families, a greater number of conflicts were observed, and the interviewees attributed this to puberty or changes in personality in the parent due to the side effects of the cancer or treatment. Often, positive changes that cancer brought to the family unit were mentioned, including a stronger sense of cohesion and an increase in joint activities such as taking walks together or family vacations.

*And then*,* very quickly*,* we drew closer together*,* that was like*,* us against cancer. Therefore*,* unnecessary conflicts were avoided right away. (I01*,* female*,* parent died of cancer)*

### Cancer impact on the adolescents

The interviewees reported a variety of changes at an individual level, such as changes in communication behaviour with family members or friends, emotional changes and influences on lifestyle, school and future decisions. Experiences with school were found to be heterogeneous. For some interviewees, school was a source of support and welcomed distraction. In contrast, others talked about difficulties concerning school, especially problems with concentration.

*I graduated that summer*,* and it was not a bad final grade*,* and I studied relatively normally for it and that was also a bit of a hold and provided stability. (I08*,* male*,* parent died of cancer)*

The most frequently reported individual changes related to emotions and mental health. The foremost concerns were the potential for individuals to develop cancer, the possibility of cancer recurrence, the risk of parental death, and the concern for parental infection with the SARS-CoV-2 virus. Many participants reported feelings of sadness and distress and attempted to suppress negative thoughts and emotions related to cancer. In contrast, the interviewees also reported positive changes in their personal development, including increased strength and responsibility and a heightened sense of gratitude.

*I wasn’t quite aware of how serious it actually was*,* and then I had moments where I kind of broke down and realised the severity*,* but then those feelings and thoughts also disappeared again. (I03*,* female*,* parent died of cancer)*

Positive lifestyle changes, such as maintaining a healthy diet and hygiene or regular attendance at cancer screenings, were also mentioned. Hobbies, especially sports, are often referred to as resources. It appeared that meeting friends was important for them to take their minds off their parents’ cancer and enjoy themselves. However, some interviewees also mentioned that they spent more time at home with their parents and minimised their contact to lower the risk of COVID-19 infection. Some young adults also reported that their parents’ cancer diagnosis had an impact on their educational path, for example, by pausing university studies to move back home or choosing a university nearby.

### Professional psychosocial support

Participant’s needs and wishes regarding psychosocial support encompass content-related preferences and practical considerations, such as the location or setting of the service and the individual providing the support. Approximately half of the participants had not received any professional psychosocial support during adolescence when their parents had cancer. Four participants were offered a psychosocial counselling programme for families, with individual appointments scheduled. Additionally, four participants received outpatient psychotherapy. However, three of them sought psychosocial support only a few years after their family had dealt with the disease. In response to the question regarding the type of support that is now considered helpful, the majority of participants cited peer-group sessions or settings that allow for personalised choices. The participants expressed a preference for a setting that facilitates the selection or combination of family sessions, individual sessions, and group sessions, with these sessions being facilitated by a psychologist, a physician, a neutral/external person (not a family member or friend), or a teacher. The location preferences of the subjects in this study varied between a neutral place, a hospital or counselling site, or a school. The present study revealed that face-to-face meetings were considered more favourable than online meetings. Only one of the participants replied that he would not want professional psychosocial support at all.

*I think what would have helped me would have been to talk to people of my age. Some kind of offer*,* because you just did not talk about it with your friends because (…) you never had the feeling that they could understand what was going on. (I11*,* female*,* parent died of cancer)*

The range of topics for which the participants expressed a desire for support included information about cancer and treatment, as well as the potential changes that cancer might bring to families. Other areas of concern included emotions, coping strategies and the possible death of the parent. However, the most significant theme was the opportunity to share their experiences with others. In terms of the group setting, the majority of interviewees expressed a preference for active elements, such as joint activities or creative components, to enhance the atmosphere. Furthermore, some participants expressed a desire for guidance on internet content, books or brochures. A lack of information about available services and the difficulty involved in accessing them were identified as key obstacles to utilising support services. It is the opinion of the participants that the most effective methods of informing adolescents about support offers and encouraging them to engage are postings, flyers, a direct approach through healthcare personnel and, most importantly, parents as gatekeepers. The interviewees also stated that support offers should ideally be initiated at the time of the initial diagnosis and should continue throughout the disease process on a regular but voluntary basis.

The participants of the study also specified their wishes for support, with a particular focus on the roles of parents and the school. The expressed desires included greater involvement from teachers and the utilisation of professional support by parents themselves.

### Advice for adolescents with a parent with cancer

When asked to offer advice to young people who have recently been affected, respondents provided a wide range of recommendations relating to how to deal with the situation individually or ideas on how to communicate and deal with parents. It is important to take time to process the situation, talk to others about it, especially about how you feel, ask questions, seek support if you feel like it, do not hide your worries and express your needs, spend time with your parents and keep in touch, try to find something positive in this situation, and most importantly, encourage your parents to seek psychosocial support themselves.

*Talk to someone who has an open ear. If you’re in a very bad state*,* do not be afraid to get professional help and*,* above all*,* advise the parents to support themselves*,* because I think the worst thing for a child is to know that the parents are not well. (I14*,* male*,* parent survived cancer)*

## Discussion

This study gathered first-hand information about the impact of a parent’s cancer diagnosis during adolescence on the lives of teenagers and young adults. It also explores retrospective views on how best to support adolescents in this situation.

The primary focus of disease-specific communication between adolescents and parents has been reported to be on diagnosis and treatment. The impact of cancer on the emotional well-being of the family unit was a less frequently addressed issue. However, the participants retrospectively expressed a desire to discuss their feelings and the strategies they used to cope with the new and challenging situation. Furthermore, the respondents expressed a desire to be informed and involved in a timely manner regarding challenging subjects, such as the death of a parent. The findings of a systematic review indicate that open family communication plays an important role in children’s coping: warm and accepting parent‒child interactions, as well as more open communication, are significantly associated with fewer emotional and behavioural problems in children [19]. Furthermore, some participants recalled experiencing ambivalence regarding the volume of information. On the one hand, the subjects expressed a desire for more information; on the other hand, they also expressed a fear of it. Previous studies have also documented inconsistencies in adolescents’ open communication. While the offspring of cancer patients often do not want to share their illness-related concerns, they still expect open communication from their parents in this regard [20]. It is therefore appropriate to involve adolescents through open but cautious communication, as well as a space to open up and share concerns. Importantly, the experiences of some of the participants in this study occurred several years ago. Nevertheless, recent studies indicate that there are still reservations, fears or difficulties in parent‒child communication when a parent has cancer [21, 22]. This finding indicates that open communication within the family, both historically and in the present, is a significant factor for the well-being of all its members. It is therefore vital that all healthcare professionals involved also focus on communication.

Some participants in the present study perceived changes such as an increase in gratitude or strength, which can be attributed to posttraumatic growth. This finding aligns with the results reported by Walczak et al. [6], who documented domains of posttraumatic growth [23] in adolescents, including enhanced appreciation of life and other individuals, personal growth and development, positive shifts in goals and priorities, and increased interest in health and improved health behaviours. Despite the absence of an explicit request for information regarding coping strategies, the interviewees reported behavioural changes that are consistent with the typical coping strategies observed in adolescents. In their report, Phillips et al. identified a number of common coping strategies employed by adolescents, including distraction to maintain normalcy, talking about the cancer, reasoning, helping the ill parent, seeking meaning and seeking social support [24]. The most frequently mentioned strategies in the present study included ‘helping the ill parent’, ‘talking about the cancer’ and ‘distraction to maintain normalcy’.

The majority of participants reported aspects of being young carers. The term ‘young carer’ is used to describe children and adolescents who provide regular and substantial care to ill or disabled family members [25]. The provision of support may take various forms, including practical assistance with activities of daily living such as shopping or domestic tasks, emotional support, and physical or personal care [26]. As the severity of parents’ illness and physical impairment increased, the interviewees naturally assumed a greater share of household responsibilities. This finding aligns with the findings of a review by Justin et al. [27], who also identified young carers in oncology and articulated the conflict between their desire to assist their family members and the need for personal time and respite that children of cancer patients may experience. There appears to be a lack of identification of young carers and recognition of their role, which should be addressed to provide them with the necessary support. Future research on young carers in oncology should therefore also investigate support needs specifically in relation to informal care.

When asked how adolescents would have preferred school to be involved, the interviewees expressed a divergence of opinion. Some respondents expressed a strong desire for cancer to be addressed in a school setting, whereas others neither wished to be asked about their parent’s health nor wanted their classmates to be aware of their personal situation. Sheehan et al. [28] explored this ambiguity in the context of a palliative disease, theorising it as a result of the struggle to manage two worlds. Some students prefer to keep the two worlds separate: the typical world of adolescent development and the challenging world of caring for a parent who is nearing the end of their life. While some participants expressed reservations, the call for greater attention to this issue in educational institutions appears justified, considering that young individuals spend up to eight hours a day in school.

With regard to psychosocial support, the majority of the respondents expressed a preference for peer group support, ideally in combination with individual or family sessions. This finding aligns with that of Morris et al. [20], who reported that the offspring of cancer patients used turning to themselves and peer support as coping strategies. The present findings are also consistent with the challenges described by Sawyer [2] during the transition to adolescence, which primarily include concerns about being ‘normal’ and the importance and influence of the peer group. Group programmes appear to be the most suitable means of addressing the needs of young people. Furthermore, the interviewees expressed a desire for enhanced access that does not rely on parents as gatekeepers. This is particularly important given that parents might underestimate the mental burden of their adolescent children [29] and that adolescents aspire to greater autonomy and thus actively seek support themselves. Furthermore, a number of respondents expressed the need for parents to seek psychosocial support. This appears to be a logical conclusion, given the established correlation between parental depression and poorer adjustment of their children [30].

Longitudinal research has demonstrated that access to psychiatric services is greater among young people who have experienced parental cancer than among those who have not [30]. In the present study, three participants stated that they had sought psychotherapeutic help later in life after having gone through the phase of parental disease without professional support. This underscores the potential preventive benefit of early initiation of psychosocial support. Overall, our findings align with previous research and recommendations on the needs of adolescents, including the provision of age-appropriate information about their parents’ cancer, support in communicating with their parents not only about the disease but also about emotions, encouragement to maintain hobbies, peer support to reduce feelings of isolation and to feel ‘normal’, and psychosocial support to cope with distress [19, 31]. The preceding findings were principally derived from samples of affected individuals who were still in their teens. Nevertheless, the validity of these findings is retained when young adults are interviewed retrospectively during this period, as was the case in the present study.

### Study limitations

It is important to interpret the findings in light of the study’s limitations. For example, the age range was broad. A narrower age range may have yielded different findings; this is a possibility that should be considered in future studies.

A further limitation of the study is the unknown effect or cognitive bias due to false memories or emotional falsification. Moreover, the periods of parental diseases partly extend back 10–15 years; therefore, experiences regarding support structures may not be up-to-date due to changes in the healthcare system. As the research was conducted in a specific geographical area, the findings cannot be generalised to other areas and cultures. Although six male participants were included in the study, there was an excess of female participants. Consequently, it is uncertain whether the results accurately reflect the needs of adolescent boys. Furthermore, the participants were predominantly from the academic sector, a group that is more likely to be drawn from a higher social class. However, the subjects were very motivated to engage in in depth, informative interviews and showed a remarkable willingness to reflect on their teenage years.

## Conclusion

This research provides important information on adolescents’ experiences with a parent diagnosed with cancer. The study emphasises the importance of transparent, age-related communication throughout the entire course of the disease. The study further demonstrates that the experiences of former adolescents provide a valuable source of insight into the positive and negative consequences of family and individual well-being, including increased cohesion, personal growth, and the presence of stress and conflict. This study provides a foundation for recommendations concerning the provision of psychosocial support services. It emphasises the importance of seeking support at an early stage and the value of connecting with other young individuals facing similar challenges, in addition to individual appointments. The present study also emphasises the importance of support services being available throughout the course of the illness, including the palliative phase. In view of the heterogeneous nature of our sample, future research could concentrate on specific gender differences and narrower age ranges. Furthermore, comprehensive research into the situation of young carers in oncology and their support needs is needed. These findings will help identify adolescents in need and provide them with comprehensive support.

Affected families appear to benefit from the supportive behavior of those around them, whether in an educational or medical setting. This is especially important for identifying, accessing and using specific professional support offers. According to the respondents in this study, the most effective methods for disseminating information about psychosocial support services and encouraging the use of these services appear to be flyers/brochures, direct contact by healthcare professionals and, above all, encouragement of parents. Furthermore, the age-typical aspiration for autonomy leads adolescents to seek access pathways that are not contingent on their parents. Further research is needed to determine how this could be implemented in detail and whether schools and teachers could play a greater role.

## Supplementary Information

Below is the link to the electronic supplementary material.

Supplementary Material 1

Supplementary Material 2

## Data Availability

No datasets were generated or analysed during the current study.

## List of abbreviations

UKE: University Medical Centre Hamburg-Eppendorf
COREQ: Consolidated criteria for reporting qualitative research
CLIMB: Children's Lives Include Moments of Bravery
LPEK: Local Psychological Ethics Committee at the Centre fpr Psychosocial Medicine
IBM SPSS V27: Statistical Package for the Social Sciences Version 27
SD: Standard Deviation
N: Number of Participants
SARS-COV-2: Severe acute respiratory syndrom coronavirus 2
COVID-19: Coronavirus disease of 2019
