# Supplementary material for: Parental cancer: communication, daily life changes and psychosocial support: a qualitative study of adolescents and young adults who experienced parental cancer during adolescence
Source: BMC Psychol. 2025 Sep 17;13:1010. doi: 10.1186/s40359-025-03396-3 (PMC12442279; doi:10.1186/s40359-025-03396-3)
Supplement: Supplementary file 1 — Supplementary Material 1 [file 40359_2025_3396_MOESM1_ESM.docx]

**Interview guide**

Thank you for agreeing to take part in our interview study.

We are conducting interviews with young people who experienced a parental cancer during their adolescence.

We would like to find out more about the burdens and needs of those affected.

In particular, we want to find out what professional psychosocial support should look like in order to really meet the needs of young people.

With the help of the reported experiences, wishes and needs that you have experienced in connection with the parental illness, we would like to expand our range of support services for families with a parent with cancer.

I am ______________________ and I am an employee at the Institute and Polyclinic for Medical

Psychology at the University Medical Centre Hamburg-Eppendorf.

The interview will take about 30 to 50 minutes. It is documented by me in handwritten notes. The audio will also be digitally recorded for later detailed analysis. I will ask you questions and address specific topics. During the interview, you will have the opportunity to freely describe the aspects that you consider important. If necessary, I will ask you more detailed questions to ensure my understanding.

If you do not wish to answer a question, that is of course not a problem.

You can also cancel the interview at any time without giving a reason. This is also possible if you have already given your consent. If you cancel the interview, the content that has already been logged and recorded will not be processed but deleted immediately.

At the beginning of the interview, I will give you your code number for later reference.

Do you have any questions about the process, content or data protection aspects?

Then let's start with the interview and the recording.

**Sociodemographic data**

1. How old are you?

2. Which country were you born in? *If other country*: How long have you lived in Germany?

3. What school-leaving qualification do you have?

4. What is your profession? What are your working hours?

5. What is your family situation and living situation? (partner, patch-work, with parents

parents, shared flat etc.)

6. Do you have children? How old are they?

7. Who was ill in your family?

8. When did the parent with the disease receive the diagnosis?

9. Tumour location and condition at initial diagnosis? localized? metastasized)?

10. Course of the disease and current condition?

11. Which treatments have taken place? Is the parent still undergoing treatment?

**Experience with parental cancer**

“In the next section, we would like to find out what that was like for you at the time. How you perceived the situation and possible changes for you and your family.”

1. Who told you about the illness? What was it like when you found out about it?

2. How was it talked about in the family? Have you actively addressed the issue yourself?

3. Where did you get information about the disease (*if nothing comes up, make offers:* Internet, family, books, brochures)?

4. Would you say that you received all the information you needed at the time to understand the illness/situation?

5. Did you ever attend a doctor's appointment of the ill parent (e.g. doctor's consultation or chemo)?

Or have you seen the parent in hospital?

6 What changes has the illness brought for the family?

7. How was it dealt with in the family? Were there differences between the family members?

8. What helped you, where did you get support?

**Support**

“In the next section, we would like to find out whether and where you received professional psychosocial support and what else you might have wanted or needed.”

1. Did you also receive professional support outside the family? (If yes, what did it look like?)

2. At what point during the illness?

3. What was it like for other family members?

4. What was the trigger, what prompted you and/or your family to seek support?

5. What type of professional support would you have wanted/needed? (From whom? And support with what exactly?)

6. Was any support offered by doctors, hospital staff or similar?

7. If you could come up with a support intervention for young people yourself, what would it look like? *If there is no response, suggest*: Individual or Family or Group; on a regular basis or only if required; Conversations, Activities, Art, Music or anything similar; Face-to-face or Online

**Current situation, Conclusion, End**

“We have now reached the end of the interview. I'll have another look to see if anything still open *(if necessary, go back to earlier questions, ask in more detail).*

Looking back, what would you say?”

1. How would you say the disease has affected you? What has it changed for you?

2. What advice would you give to children/adolescents who are newly affected by a parent’s cancer diagnosis?

3. How are you doing today? What does your life look like?

“Is there anything else from your side that you consider important that you were unable to report in the context of the questions?

Do you have any further questions to ask me?”

Thank you very much for taking part in our study.
